# Identification of Genetic Alterations, as Causative Genetic Defects in Long QT Syndrome, Using Next Generation Sequencing Technology

**DOI:** 10.1371/journal.pone.0114894

**Published:** 2014-12-10

**Authors:** Oscar Campuzano, Georgia Sarquella-Brugada, Irene Mademont-Soler, Catarina Allegue, Sergi Cesar, Carles Ferrer-Costa, Monica Coll, Jesus Mates, Anna Iglesias, Josep Brugada, Ramon Brugada

**Affiliations:** 1 Cardiovascular Genetics Center, University of Girona-IdIBGi, Girona, Spain; 2 Arrhythmia Unit, Hospital Sant Joan de Déu, University of Barcelona, Barcelona, Spain; 3 Gendiag SL, Barcelona, Spain; 4 Cardiology Service, Hospital Josep Trueta, Girona, Spain; Sackler Medical School, Tel Aviv University, Israel

## Abstract

**Background:**

Long QT Syndrome is an inherited channelopathy leading to sudden cardiac death due to ventricular arrhythmias. Despite that several genes have been associated with the disease, nearly 20% of cases remain without an identified genetic cause. Other genetic alterations such as copy number variations have been recently related to Long QT Syndrome. Our aim was to take advantage of current genetic technologies in a family affected by Long QT Syndrome in order to identify the cause of the disease.

**Methods:**

Complete clinical evaluation was performed in all family members. In the index case, a Next Generation Sequencing custom-built panel, including 55 sudden cardiac death-related genes, was used both for detection of sequence and copy number variants. Next Generation Sequencing variants were confirmed by Sanger method. Copy number variations variants were confirmed by Multiplex Ligation dependent Probe Amplification method and at the mRNA level. Confirmed variants and copy number variations identified in the index case were also analyzed in relatives.

**Results:**

In the index case, Next Generation Sequencing revealed a novel variant in *TTN* and a large deletion in *KCNQ1*, involving exons 7 and 8. Both variants were confirmed by alternative techniques. The mother and the brother of the index case were also affected by Long QT Syndrome, and family cosegregation was observed for the *KCNQ1* deletion, but not for the *TTN* variant.

**Conclusions:**

Next Generation Sequencing technology allows a comprehensive genetic analysis of arrhythmogenic diseases. We report a copy number variation identified using Next Generation Sequencing analysis in Long QT Syndrome. Clinical and familiar correlation is crucial to elucidate the role of genetic variants identified to distinguish the pathogenic ones from genetic noise.

## Introduction

The long QT syndrome (LQTS) is an inherited cardiac disorder characterized by prolonged QT interval on the surface electrocardiogram (ECG). It affects 1/2500 individuals, causing lethal ventricular tachy­cardias (VT), *torsades de pointes* (TdP) and sudden cardiac death (SCD) [1]. These events can be triggered by physical or emotional stress, but in some individuals they may occur during periods of sleep or rest. However, there is important phenotypic heterogeneity [2].

Genetic studies have shown that LQTS is caused by pathogenic mutations in 15 genes encoding cardiac ion channels or membrane adaptors (*KCNQ1, KCNH2, SCN5A*, *ANK2, KCNE1, KCNE2, KCNJ2, CACNA1C, CAV3, SCN4B, AKAP9, SNTA1, RYR2, KCNJ5* and *SCN1B*) [3]. Pathogenic mutations identified in the *KCNQ1* and *KCNH2* genes as well as the sodium channel, encoded by *SCN5A,* are responsible for nearly 80% of all clinically diagnosed cases. All the other genes together explain less than 5% of LQTS cases. Recently, large intragenic deletions and duplications have been reported in LQTS families, suggesting that the cause of disease in some patients could be the presence of copy number variants (CNVs) affecting the major genes for LQTS. Detection rate for CNVs among LQTS patients, mutation-negative by traditional analysis, seem to be around 2–11.5% [4]–[7]. Other unknown genetic causes might be responsible for the remaining LQTS cases, such as mutations in non-coding regions and novel mutations in as yet unknown genes [6], [8].

Currently, most genetic studies focus on the analysis of the main genes associated with LQTS, following current clinical guidelines for LQTS [9]. All these studies use conventional Sanger sequencing. Because of its high cost, a comprehensive genetic analysis has not regularly been performed in LQTS for all genes. In recent years, Next-Generation Sequencing (NGS) has emerged as a revolutionary technology which enables the generation of high amount of genetic data [10]. This massive amount of information has triggered the development of potent bioinformatic tools to help interpret potential causality implications [11], [12].

The goal of our study was to identify the genetic alteration that could explain the LQTS in our family. Because of substantial percentage of LQTS cases without genetic diagnose after screening of all known LQTS genes, we used a NGS custom panel to screen the main genes associated with SCD.

## Materials and Methods

### Clinical evaluation

All relatives included in our study were clinically evaluated at our Pediatric Arrhythmia Unit. Complete clinical evaluation, including electrocardiogram (ECG), transthoracic echocardiogram (ECHO), 24-hour ECG Holter recording and exercise test was performed in index case and all relatives. This study was approved by the Ethics Committee of Hospital Josep Trueta (Girona, Spain) and conforms to the principles outlined in the Declaration of Helsinki. All individuals signed a written informed consent to participate in the study. Informed consent of all patients was obtained in accordance with international review board guidelines of Hospital Josep Trueta and Universitat of Girona (Girona, Spain).

### DNA sample

Genomic DNA was extracted with Chemagic MSM I from whole blood (Chemagic human blood). DNA samples were checked in order to assure quality and quantify before processing to get the 3µg needed for the NGS strategy. DNA integrity was assessed on a 0,8% agarose gel. Spectrophotometric measurements are also performed to assess quality ratios of absorbance; dsDNA concentration is determined by fluorometry (Qubit, Life Technologies). DNA sample was fragmented by Bioruptor (Diagenode). Library preparation was performed according to the manufacturer’s instructions (SureSelect XT Custom 0.5–2.9 Mb library, Agilent Technologies, Inc). After capture, the indexed library was sequenced in a six-sample pool cartridge. Sequencing process was developed on MiSeq System (Illumina) using 2×150 bp reads length.

### Custom Resequencing panel

We selected 55 genes, the most prevalent involved in SCD-related pathologies, according to available scientific literature. The genomic coordinates corresponding to these 55 genes (Table 1) were designed using the tool eArray (Agilent Technologies, Inc.). All the isoforms described at the UCSC browser were included at the design. The final size was 432,512 kbp of encoding regions and ´UTR boundaries. The coordinates of the sequence data is based on NCBI build 37 (UCSC hg19).

**Table 1:** List of the 55 SCD-related genes included in our panel and its association with the disease.

DISEASE	GENES
Brugada Syndrome	*CACNA1C, CACNB2, GPD1L, HCN4, SCN5A*
Long QT Syndrome	*ANK2, CACNA1C, CAV3, KCNE1, KCNE2, KCNH2, KCNJ2, KCNQ1, RYR2, SCN4B, SCN5A*
Short QT Syndrome	*CACNA1C, CACNB2, KCNH2, KCNJ2, KCNQ1*
Catecholaminergic Polymorphic Ventricular Tachycardia	*CASQ2, KCNJ2, RYR2*
Hypertrophic Cardiomyopathy	*ACTC1, ACTN2, CAV3, CSRP3, GLA, JPH2, LAMP2, LDB3, MYBPC3, MYH6, MYH7, MYL2, MYL3, MYOZ2, PDLIM3, PLN, PRKAG2, RYR2, TCAP, TNNC1, TNNI3, TNNT2, TPM1, TTN, VCL*
Dilated Cardiomyopathy	*ACTC1, ACTN2, CAV3, CRYAB, CSRP3, DES, DMD, DSC2, DSG2, DSP, EMD, LAMP2, LDB3, LMNA, MYBPC3, MYH6, MYH7, PKP2, PLN, SCN5A, SGCD, TAZ, TCAP, TNNC1, TNNI3, TNNT2, TPM1, TTN, VCL*
Arrhythmogenic Right Ventricular Cardiomyopathy	*DES, DSC2, DSG2, DSP, JUP, LMNA, PKP2, PLN, TGFB3, TTN*

### Bioinformatics

The secondary bioinformatic analysis of the data obtained includes a first step trimming of the FAST-Q files. The trimmed reads are then mapped with GEM II and output is joined and sorted and uniquely and properly mapping read pairs are selected. Finally, variant call over the cleaned BAM file is performed with SAMtools v.1.18, GATK v2.4 to generate the first raw VCF files. Variants are annotated with dbSNP IDs, Exome Variant Server and the 1000 Genomes browser, in-home database IDs and Ensembl information, if available.

Tertiary analysis is then developed. For each genetic variation identified, allelic frequency was consulted in Exome Variant Server -EVS- (http://evs.gs.washington.edu/EVS/) and 1000 genomes database (http://www.1000genomes.org/). In addition, Human Gene Mutation Database -HGMD- (http://www.hgmd.cf.ac.uk/ac/index.php) was also consulted to identify pathogenic mutations previously reported. *In silico* pathogenicity of novel genetic variations were consulted in CONDEL software (CONsensus DELeteriousness scores of *missense* SNVs) (http://bg.upf.edu/condel/), and PROVEAN (Protein Variation Effect Analyzer) (http://provean.jcvi.org/index.php). Alignment among species was also performed for these novel variations using UniProt database (http://www.uniprot.org/).

Regarding CNV identification using NGS data, a new methodology was developed. Our approach focused on capturing significant differences between expected normalized coverage and obtained normalized coverage for a given sample in the region of interest. We normalized the raw coverage by the amount of DNA yielded for each sample in the MiSeq run. The log2 ratio data between samples was evaluated. Detection of losses and gains were based on those genomic coordinates with a log2 ratio near the stringent ratio cut-offs for duplication or deletion (less than −1.0 or greater than 0.6, respectively). Several samples were analyzed to corroborate similar levels of coverage between samples.

### Genetic confirmation

#### Sanger sequencing

Non-common (Minor Allele Frequency –MAF- <1%) genetic variants were confirmed by Sanger method. First, polymerase chain reaction (PCR) was performed. PCR products were purified using ExoSAP-IT (USB Corporation, Cleveland, OH, USA), and the analysis of the exonic and intron-exon regions was performed by direct sequencing (Genetic Analyzer 3130XL, Applied Biosystems, CA, USA) with posterior SeqScape Software v2.5 (Life Technologies) analysis comparing obtained results with the reference sequence from hg19. Each sample underwent a genetic study of corresponding genes (NCBI -National Center for Biotechnology Information-, http://www.ncbi.nlm.nih.gov/) (*TTN* NM_133378). Familial cosegregation of rare genetic variants was also performed using Sanger technology.

#### Multiplex Ligation dependent Probe Amplification

The CNV detected by NGS was confirmed by Multiplex Ligation dependent Probe Amplification (MLPA), using the probemix SALSA MLPA P114-B2 Long-QT (MRC-Holland, Amsterdam, the Netherlands). This kit contains 17 probes for the *KCNQ1* gene, 16 probes for *KCNH2*, 4 probes for *KCNE1* and 2 probes for *KCNE2*. The MLPA DNA detection and quantification were carried out according to the manufacturer’s protocol (MRC-Holland, Amsterdam, The Netherlands). After the multiplex PCR reaction, electrophoresis was performed using the ABI3130xl Genetic Analyzer (Applied Biosystems, CA, USA). Data was collected and analysed with Coffalyser. Net software (MRC-Holland). Significantly (>30%) decreased or increased signals in the patient sample relative to controls were considered as deletions or duplications, respectively. Familial cosegregation of CNVs was also performed using MLPA.

#### Sequencing of cDNA

The deletion of exons 7 and 8 of *KCNQ1* was also confirmed at the mRNA level in both the brother and the mother of the proband (index case refused analysis, and the healthy father was analysed as a control). Total RNA was isolated with the QIAamp RNA Blood Mini Kit and converted to cDNA with the QuantiTect Reverse Transcription Kit (both from Qiagen, California, USA). Afterwards, amplicon spanning from exon 6 to 9 of *KCNQ1* of the cDNA was generated by PCR using the primers 5′ACCCTGTACATCGGCTTCC3′ and 5′GGGTGACAGCAGAGTGTGG3′. PCR products were purified and sequenced (with the same primers) according to the abovementioned protocol for Sanger sequencing.

## Results

### Clinical

**Figure 1:**
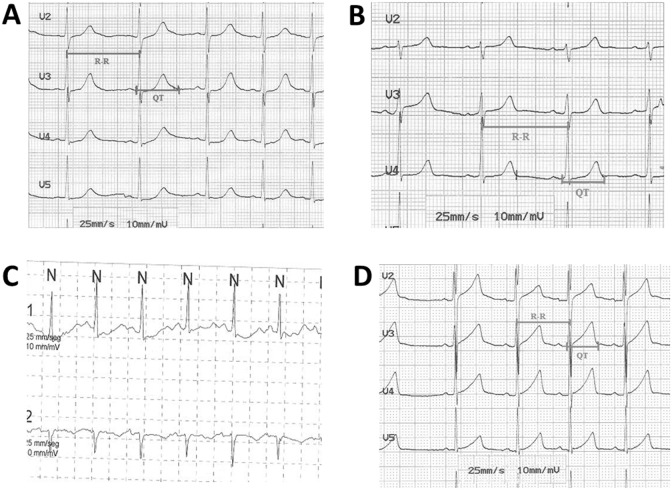
ECG of family members. (A) Twelve-lead ECG of index case. The ECG shows QTc of 500 ms. (B) Twelve-lead ECG of mother’s index case. The ECG shows a normal QTc, and (C) a LQT during tachycardia registered by Holter. (D) Twelve-lead ECG of brother’s index case. The ECG shows QTc of 485 ms.

The proband (female, 14 years old) was seen in our Paediatric Arrhythmia Unit for abnormal ECG performed in pre-exercise screening. She was asymptomatic for the cardiac point of view. Baseline ECG showed a corrected QT interval (QTc) using Bazhett formula of 500 ms (Fig. 1A). She was on no medication and had no ionic alteration which could explain the prolonged QT. Echocardiography was normal. 24-hour ECG Holter showed no arrhythmic events, and exercise test showed long QT interval.

Both parents were studied. The proband’s father had a normal ECG, 24-hour ECG Holter and exercise test. The proband’s mother had a normal QTc interval at baseline ECG but with paradoxal response to tachycardisation (Fig. 1B, 1C). The 10 year-old brother had prolonged QTc (485 ms) interval at baseline ECG (Fig. 1D). Affected patients were treated with beta-blockers.

### NGS analysis

**Figure 2:**
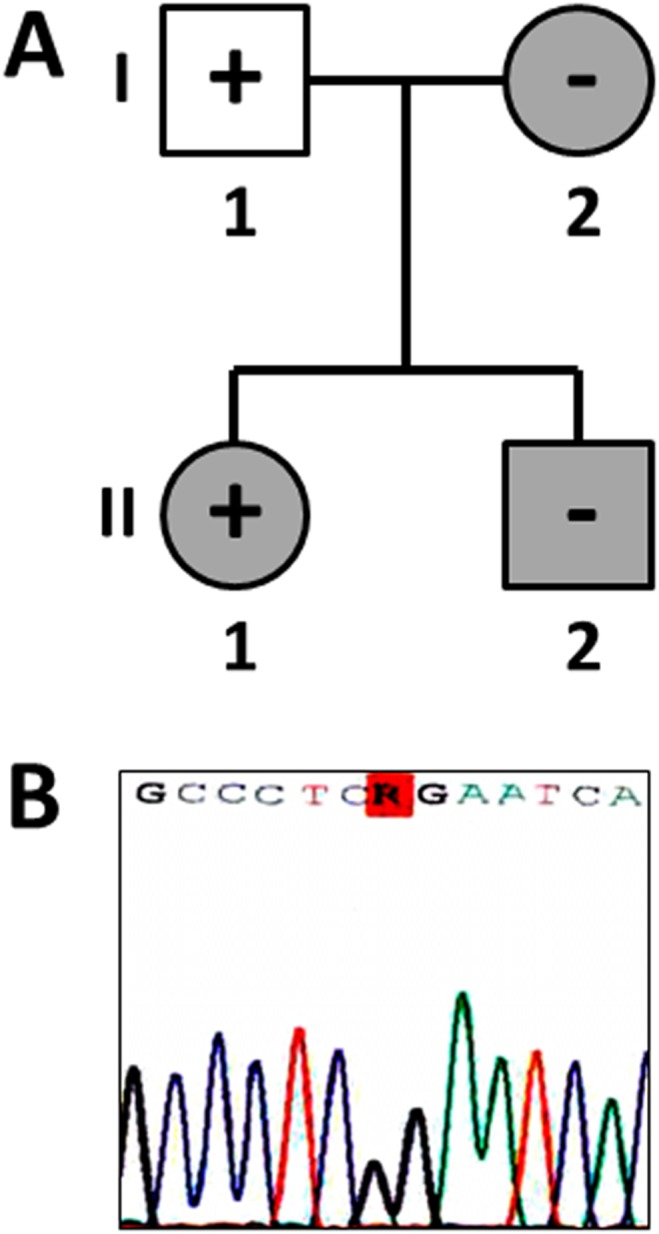
Pedigree and electropherogram. (A) Index case is II.1. White round/squares indicate healthy status after clinical evaluation. Grey round/squares indicate LQTS after clinical evaluation. Plus sign indicates carrier of genetic variation. Minus sign indicates non-carrier of the genetic variation. (B) Electropherogram of the genetic variation identified (p.R20729G_*TTN*).

We analyzed 55 genes previously associated with SCD (Table 1). After the NGS process and the application of bioinformatics pipeline, the call rate ranged from 99,6% to 98,92% at 20x and 100x respectively in this sample. We selected the Non Synonymous (NS) variants with a MAF<1% in the EVS for its conventional Sanger sequencing confirmation. Only one single nucleotide variant (SNV) was confirmed in the index case, the *TTN* gene (p.R20729G). This novel variant is consequence of a nucleotide change of A to G (c.62185A>G). The genetic variation was not previously identified in locus specific databases, considered therefore a novel GVUS. It was predicted *in silico* as pathogenic in all databases consulted. In addition, alignment between species showed a high level of conservation. However, family segregation showed that only the index case’s father carried the same genetic variation (Fig. 2).

**Figure 3:**
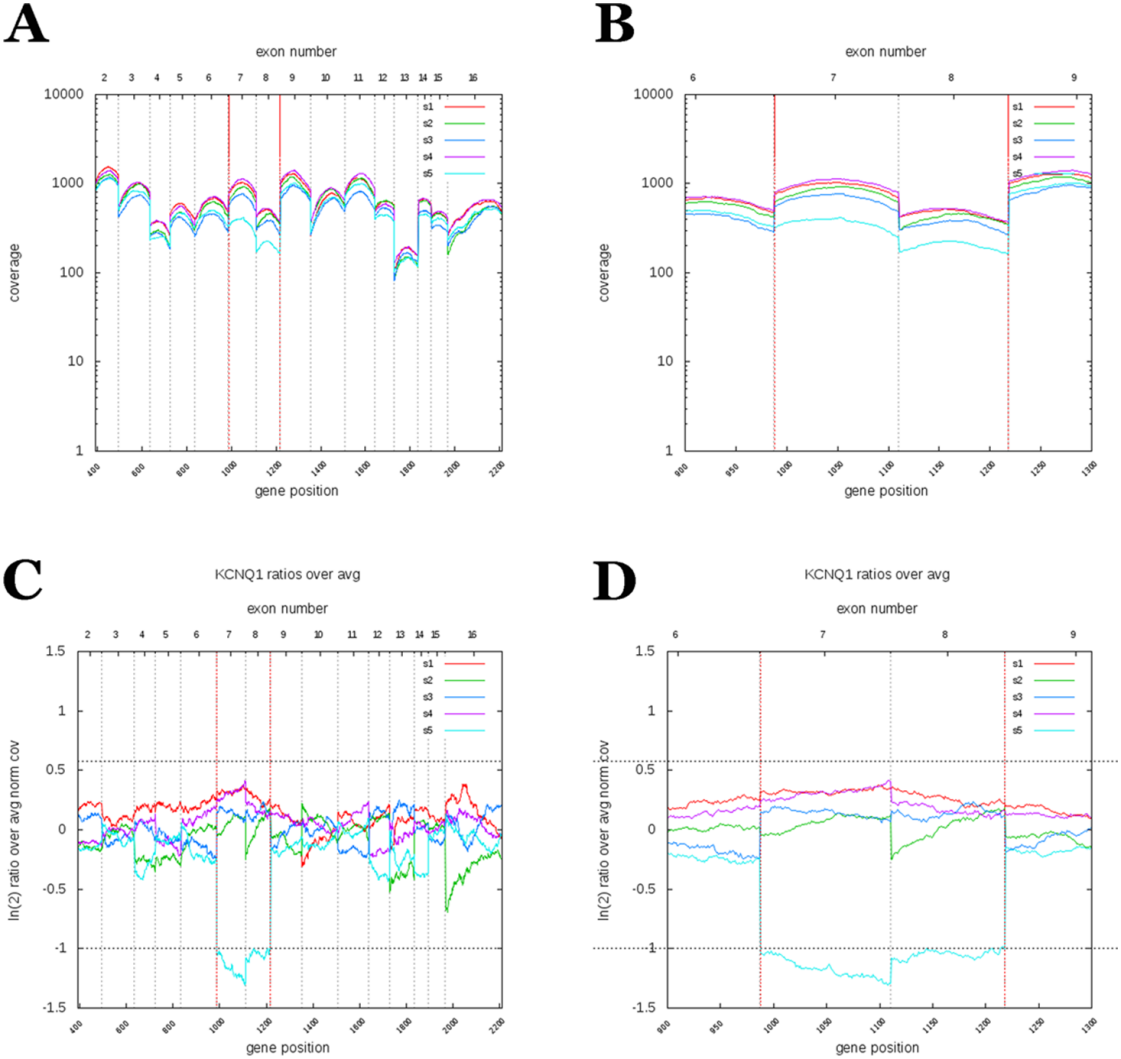
NGS data showing CNV in the *KCNQ1* gene. (A) Coverage of all exons in the *KCNQ1* gene of several samples. (B) Detail coverage of exons 7 and 8 in several samples. (C) Normalized raw coverage of exons 7 and 8 showing a deletion in comparison to all other exons of the same gene. (D) In detail, normalized raw coverage of exons 7 and 8.

**Figure 4:**
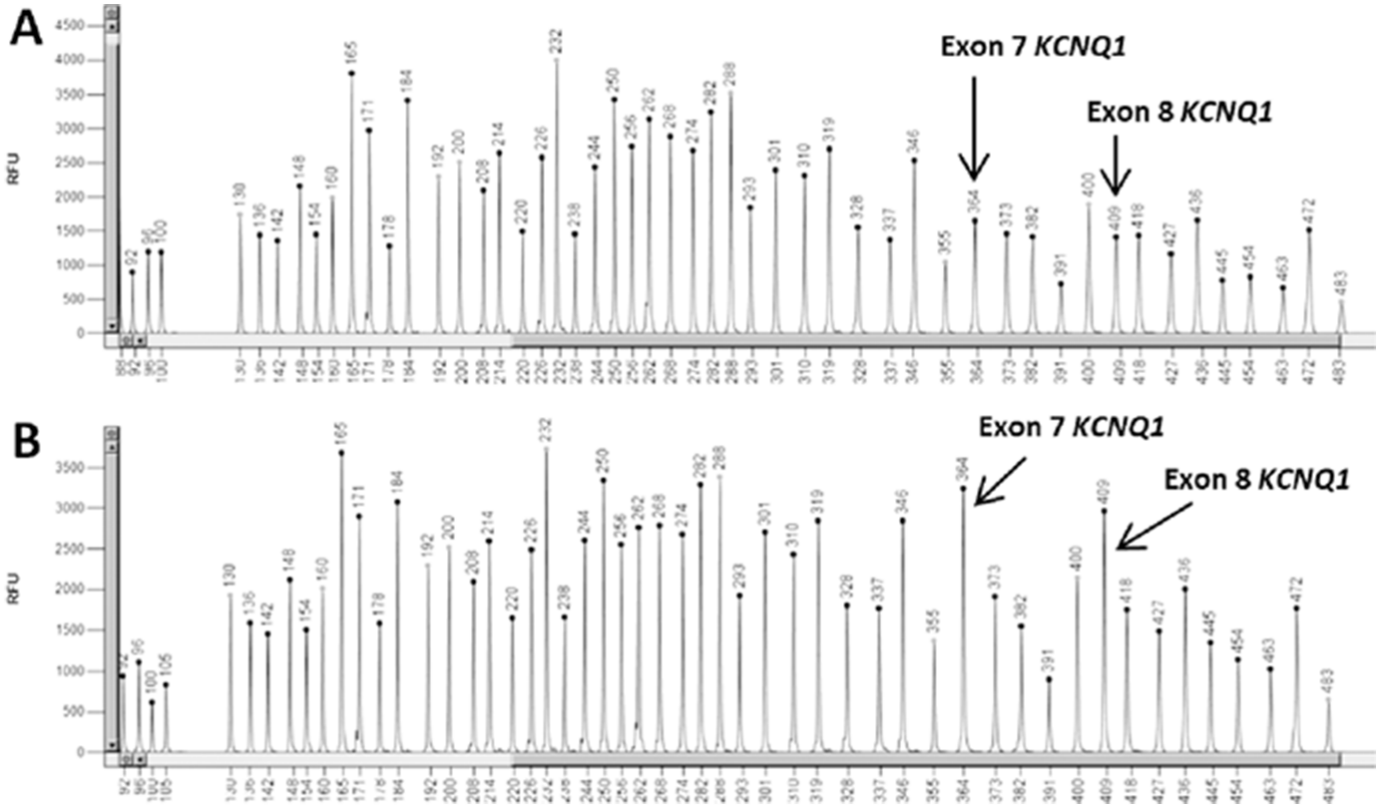
MLPA capillary electrophoresis pattern. (A) index case and (B) her healthy father, both analysed with SALSA MLPA probemix P114-B2 Long QT. Comparing both profiles, the patient’s deletion of exons 7 and 8 of the *KCNQ1* gene can be appreciated.

**Figure 5:**
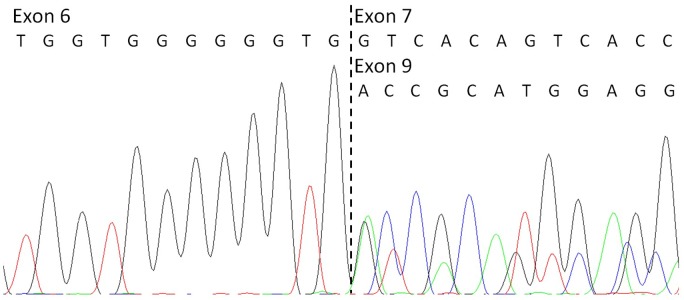
Partial electropherogram of the sequence of the cDNA of the proband’s affected brother. It confirms the deletion of exons 7 and 8 of *KCNQ1* at mRNA level.

On the other hand, NGS analysis revealed a deletion of exons 7 and 8 in the *KCNQ1* gene (Fig. 3). The raw coverage normalization showed that pooled samples were comparable in terms of coverage and no major biases between samples were found (average normalized coverage is 6.7 with sd 0.11 yielding a cv of 1.7%; average sd of normalized coverage is.60 with sd 0.02 yielding a cv of 4.1%). Then, the analysis of corrected log2 ratio coverage by genomic position for each sample was performed. The corrected log2 ratios fit a Gaussian distribution. A baseline from all pool was inferred and each sample was compared with this prediction. The deviated exons from this baseline were labelled as duplications or deletions. The analysis showed an intense signal over these two exons with more than 6 standard deviations from the mean (log2 mean ratio for this signal is −1,1±0,09 sd). This CNV alteration was confirmed by MLPA (Fig. 4). Family segregation studies revealed that the brother and the mother of the proband (both affected by LQTS) shared the same CNV, while the father’s MLPA pattern was normal. The deletion of exons 7 and 8 of *KCNQ1* was also confirmed in the brother and the mother of the proband at the mRNA level (Fig. 5).

## Discussion

The LQTS is a SCD-related channelopathy of genetic origin. According to current guidelines, when there is a suspicion of LQTS, the genetic analysis using Sanger technology of the three main genes associated with the disease is recommended. It is established that this was a cost-effective approach, until recently, with the advent of NGS technology, which makes the analysis, faster, more extensive and cost effective. NGS data could also be used to analyse CNV alterations, though pipeline bioinformatics analyses are not yet well developed. Thus, to date, few reports showing CNV in LQTS families have been published. We performed a thorough analysis covering all exons, and utilizing normalized data. Our novel approach revealed a deletion of exons 7 and 8 in the *KCNQ1* gene. After deep analysis of the protein structure, the deletion was considered as probably pathogenic. In our family, there was complete cosegregation of LQTS phenotype with the *KCNQ1* deletion, and also complete penetrance. This CNV, confirmed by MLPA method and at mRNA level, is considered extremely rare, as overlapping deletions have only been described in one work based on Asian population, and with a frequency of 0.12% [13]. A very similar CNV was previously reported by Barc et al. [6] in a patient with LQTS and without single nucleotide mutations in genes *KCNQ1*, *KCNH2* and *SCN5A*. In that family, the deletion was also identified in the proband’s father, who had an undetermined phenotype. This fact may be due to the incomplete penetrance often observed in LQTS families. Other CNVs within or including the *KCNQ1* gene have also been described in LQTS patients [4]–[6], [14]. All together, these results suggest the deletion of exons 7 and 8 in gene *KCNQ1* may be the cause of the LQTS in our family. CNVs in the *KCNH2* gene have also been reported in association with LQTS [4], [6], [7], [15]–[17]. Considering previously published series CNVs in *KCNQ1* and *KCNH2*, account for 2–11.5% of LQTS cases [4]–[7]. This percentage seems to be higher than the frequency of single nucleotide pathogenic variants in minor genes related to LQTS.

In addition, after NGS analysis, we identified a novel genetic variation in titin protein (p.R20729G_*TTN*) not reported in international databases, so far. The TTN variant was predicted as pathogenic by i*n* *silico* tools, alignment showed high conservation between species, and aminoacid change confirms a substitution of R (Arg –polar with positive charge-) to G (Gly –polar without charge-). All these facts suggest a potentially pathogenic role. Genetic studies using NGS technology reveals much higher prevalence of previously TTN-associated variants, disputing their possible causality [18]. Hence, recent studies recommend the use of several genetic tools in order to clarify its role in causing the disease, especially for clinical diagnosis [11], [12]. Though no clinical association between any structural gene and LQTS has been yet identified to our knowledge. Especially important was the fact that the variation did not segregate with the affected family members; two LQTS affected members did not carry the genetic variation. This fact confirmed that this novel variation could be discarded as a potential cause of LQTS, at least in our family. This reinforces the importance of family segregation in clinical genetics. If not available, the role of a GVUS in causing disease should be taken with great caution.

Our index case and family members diagnosed by LQTS were placed under beta-blockers, recommended exercise restriction, and provided with a list of QT prolonging drugs list, following current guidelines [9]. In these recommendations, genetic analysis is considered one of the parameters to consider in clinical diagnosis, only when a pathogenic mutation has been identified.

In summary, in familial LQTS, despite that current clinical guidelines recommend genetic analysis restricted to the main genes associated with LQTS, we provide the evidence that NGS technology can be used efficiently to analyse the rest of the genes associated with the disease. Phenotype interpretation of all these variants remains as the main challenge for its clinical translation. Despite several bioinformatic tools helps to clarify the role of genetic variants, we consider that family segregation should be the first item to be considered and analysed. Multidisciplinary teams including cardiologist and geneticist specialized in SCD related pathologies are crucial to perform an accurate clinical interpretation of all genetic data obtained, and provide helpful genetic counselling.
